# Identification of DNA-PKcs as a primary resistance factor of TIC10 in hepatocellular carcinoma cells

**DOI:** 10.18632/oncotarget.16073

**Published:** 2017-03-10

**Authors:** Long Cheng, Yuan-yuan Liu, Pei-Hua Lu, Yi Peng, Qiang Yuan, Xin-shi Gu, Yong Jin, Min-Bin Chen, Xu-ming Bai

**Affiliations:** ^1^ Department of Interventional Radiology, the Second Affiliated Hospital of Soochow University, Soochow University, Suzhou, China; ^2^ Department of Oncology, Kunshan First People's Hospital Affiliated to Jiangsu University, Kunshan, China; ^3^ Department of Medical Oncology, Wuxi People's Hospital Affiliated to Nanjing Medical University, Wuxi, China; ^4^ Department of Radiotherapy, Hubei Cancer Hospital, Wuhan, China

**Keywords:** hepatocellular carcinoma (HCC), TIC10, DNA-PKcs, TRAIL and chemosensitization

## Abstract

The current study tested the anti-hepatocellular carcinoma (HCC) cell activity of TIC10, a first-in-class small-molecule tumor necrosis (TNF)-related apoptosis-inducing ligand (TRAIL) inducer. TIC10 exerted potent anti-proliferative and pro-apoptotic actions in primary and established human HCC cells. TIC10 blocked Akt-Erk activation, leading to Foxo3a nuclear translocation, as well as TRAIL and death receptor-5 (DR5) transcription in HCC cells. We propose that DNA-PKcs is a major resistance factor of TIC10 possibly via inhibiting Foxo3a nuclear translocation. DNA-PKcs inhibition, knockdown or mutation facilitated TIC10-induced Foxo3a nuclear translocation, TRAIL/DR5 expression and cell apoptosis. Reversely, exogenous DNA-PKcs over-expression inhibited above actions by TIC10. *In vivo*, oral administration of TIC10 significantly inhibited HepG2 tumor growth in nude mice, which was further potentiated with Nu7026 co-administration. Thus, TIC10 shows promising anti-HCC activity, alone or together with DNA-PKcs inhibitors.

## INTRODUCTION

The prognosis and five-year survival of hepatocellular carcinoma (HCC) patients are often poor, yet the incidence is rising [1, 2]. Groups including ours [3–5] are dedicated to indentifying novel therapeutic targets of HCC, and to developing molecularly-targeted agents [6–8].

TRAIL, or tumor necrosis (TNF)-related apoptosis-inducing ligand, selectively kills cancer cells [9]. Yet, the clinical applications of TRAIL and TRAIL-related agents are facing several drawbacks, including the high-costing, short half-life and lack of long-lasting efficiency of these agents [9]. Recently, TIC10 (TRAIL-inducing compound 10, also named as ONC21), a first-in-class small-molecule TRAIL inducer, was developed [10–13]. Preclinical studies have demonstrated that TIC10 could be a novel and promising anti-cancer agent, which efficiently kills various cancer cells [10, 13–18]. TIC10 blocks Akt and Erk signalings, leading to Foxo3a nuclear translocation, which dictates TRAIL and death receptor-5 (DR5) transcription [11, 12, 19]. This would eventually activate TRAIL-mediated apoptosis pathway to kill cancer cells [11, 12, 19]. To our best knowledge, the potential effect and possible underlying mechanisms of TIC10 against human HCC cells were studied.

The other important aim of this study is identify TIC10 resistance factors. We focused on the possible function of DNA activated protein kinase (DNA-PK) catalytic subunit (DNA-PKcs). DNA-PKcs and two Ku hetero-dimer (Ku-70 and Ku-80) form DNA-PK complex [20, 21]. It has been shown that DNA-PKcs expression is significantly upregulated in HCC and other cancers, which is often associated with a poor clinical outcome [22]. For example, Evert *et al*., has shown that DNA-PKcs upregulation might be important for human hepatocarcinogenesis and a putative prognostic marker [23]. Meanwhile, DNA-PKcs could also serve as a HCC tissue biomarker that predicts response to treatment and survival [22]. Intriguingly, DNA-PKcs not only can protect cancer cells from harmful DNA insults, but could also possess other functions to promote cancer cell survival and proliferation [24]. Inhibition, silence and loss-of-function mutation of DNA-PKcs were shown to inhibit a number of cancer cells [25–31]. In this study, we propose that DNA-PKcs could be a primary resistant factor of TIC10 in HCC cells.

## RESULTS

### TIC10 inhibits HCC cell proliferation *in vitro*

**Figure 1:**
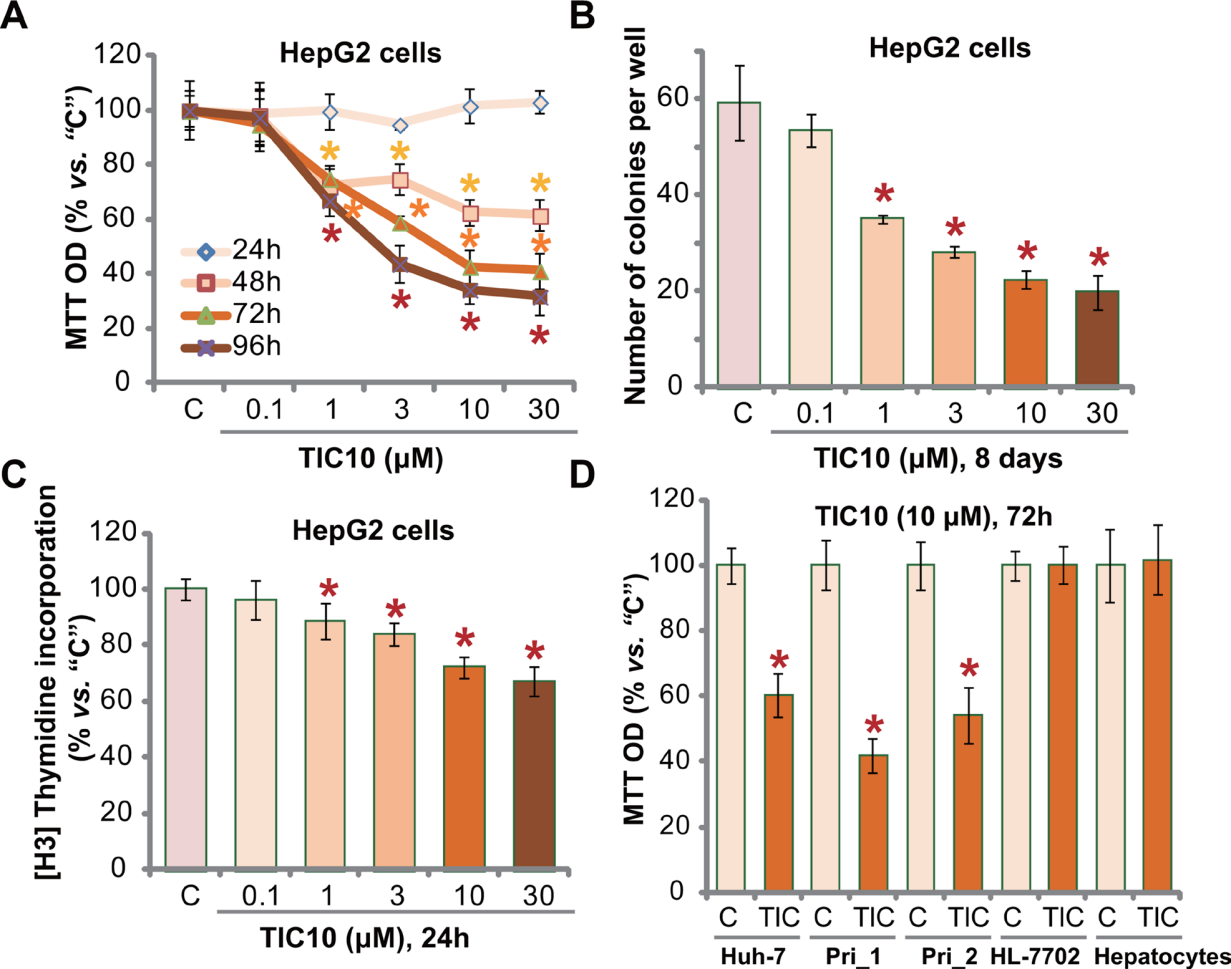
TIC10 inhibits HCC cell proliferation *in vitro* Established HCC cell lines, HepG2 (**A**–**C**) and Huh-7 (**D**), primary human HCC cells (D, “Pri_1/Pri _2”), as well as HL-7702 human hepatocytes (D) and primary human adult hepatocytes (“Hepatocytes”, D), were either left untreated (“C”, same for all figures), or treated with applied concentration of TIC10 (0.1–30 μM), cells were then cultured in conditional medium for applied time; Cell proliferation was tested by MTT assay (A and D), clonogenicity assay (B) and [H^3^] Thymidine incorporation assay (C). Experiments in this figure were repeated for five times, with similar results obtained. *n* = 5 for each repeat. Bars stand for mean ± SD. *p < 0.05 vs. group “C”.

In order to test the potential effect of TIC10 *in vitro*, HCC HepG2 cells, cultured in FBS-containing complete medium, were treated with TIC10 at gradually-increasing concentrations: from 0.1 to 30 μM. MTT assay was first applied to test cell proliferation. Results in Figure 1A demonstrated that TIC10, at 1–30 μM, dose-dependently inhibited proliferation of HepG2 cells. TIC10, at 0.1 μM, failed to alter cell proliferation (Figure 1A). Further, TIC10 displayed a time-dependent response in suppressing HepG2 cell proliferation (Figure 1A). It would require at least 48 hours for TIC10 (1–30 μM) to exert significant anti-proliferative action (Figure 1A). Clonogenicity assay results showed that TIC10 treatment decreased the number of proliferative HepG2 colonies, again in a dose-dependent manner (Figure 1B). Meanwhile, as shown in Figure 1C, 1-30 μM of TIC10 significantly decreased the [H^3^] Thymidine incorporation in HepG2 cells, which further confirmed its anti-proliferative activity.

We also tested the effect of TIC10 in other HCC cells. In both Huh-7 (another established HCC cell line [32, 33]) and primary human HCC cells (two lines, “Pri_1/Pri _2”, see Method), treatment with TIC10 (10 μM, 72 hours) similarly inhibited cell proliferation (Figure 1D). Remarkably, the very same TIC10 treatment (10 μM, 72 hours) failed to inhibit the proliferation (MTT OD) of non-cancerous HL-7702 hepatocytes ([5, 34]) and primary human adult hepatocytes (Figure 1D). Collectively, these results demonstrate that TIC10 selectively inhibits human HCC cell proliferation *in vitro*.

### TIC10 induces TRAIL and DR5 expression, provokes apoptosis in HCC cells

**Figure 2:**
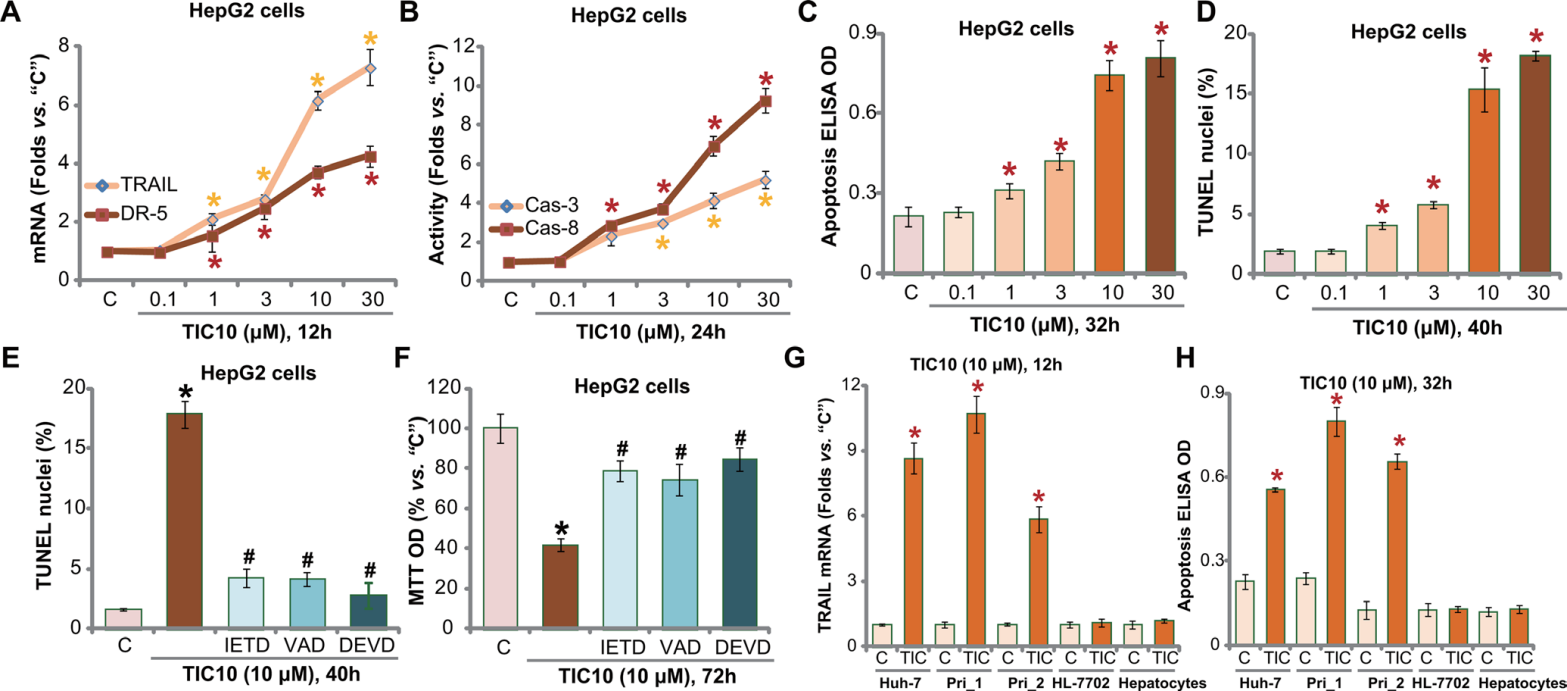
TIC10 induces TRAIL and DR5 expression, provokes apoptosis in HCC cells HepG2 cells (**A**–**F**) Huh-7 cells (G and H), primary human HCC cells (**G** and **H**, “Pri_1/Pri _2”), as well as HL-7702 human hepatocytes (G and H) and primary human adult hepatocytes (“Hepatocytes”, G and H), were treated with applied concentration of TIC10 (0.1–30 μM), cells were then cultured in conditional medium for applied time; TRAIL/DR5 mRNA expression (A and G) and capase-3/−8 (“Cas-3/−8”) activation (B) were tested; Apoptosis was tested by listed assays (C, D and H). HepG2 cells, pretreated for 1 hour with 50 μM of z-IETD-fmk (“IETD”), z-DEVD-fmk (“DEVD”) or z-VAD-fmk (“VAD”), were treated with TIC10 (10 μM) for applied time; Cell apoptosis (E, TUNEL assay) and proliferation (F, MTT assay) were tested. Experiments in this figure were repeated for four times, with similar results obtained. *n*=5 for each repeat. Bars stand for mean ± SD *p < 0.05 vs. group “C”. ^#^*p* < 0.05 vs. TIC10 only (E and F).

Proliferation inhibition by TIC10 in HCC cells could be due to apoptosis. As discussed, TIC10 induces TRAIL and DR5 expression to promote cell apoptosis [10–14, 35]. We thus tested its potential effect in HCC cells. As demonstrated, treatment with TIC10 in HepG2 cells indeed induced mRNA expression of TRAIL and DR5 (Figure 2A). Consequently, caspase-8 and downstream caspase-3 were activated (Figure 2B) in TIC10-treated HepG2 cells. These results imply TRAIL-dependent apoptosis pathway activation following TIC10 treatment. Indeed, TIC10, at 1-30 μM, potently induced HepG2 cell apoptosis, which was evidenced by increase of Histone DNA ELISA OD (Figure 2C) and parentage of TUNEL nuclei (Figure 2D). The pro-apoptotic activity by TIC10 was dose-dependent (Figure 2A–2D).

Next, several caspase inhibitors were applied, including the caspase-8 inhibitor z-IETD-fmk, the caspase-3 inhibitor z-DEVD-fmk and the general caspase inhibitor z-VAD-fmk. As demonstrated, co-treatment with these caspase inhibitors remarkably inhibited TIC10 (10 μM)-provoked HepG2 cell apoptosis (TUNEL assay, Figure 2E). More importantly, TIC10-induced proliferation inhibition was also significantly attenuated with application of these caspase inhibitors (Figure 2F). These results indicate that TIC10 provokes caspase-8-dependent apoptosis to inhibit HepG2 cell proliferation.

In both Huh-7 cells and the primary human HCC cells, treatment with TIC10 (10 μM) similar induced TRAIL mRNA expression (Figure 2G) and cell apoptosis (Figure 2H). On the other hand, no significant TRAIL induction and apoptosis activation were noticed in TIC10-treated HL-7702 hepatocytes and primary human adult hepatocytes (Figure 2G and 2H). Collectively, these results demonstrate that TIC10 induces TRAIL and DR5 expression, and provokes HCC cell apoptosis.

### DNA-PKcs could be a primary resistance factor of TIC10 in HCC cells

**Figure 3:**
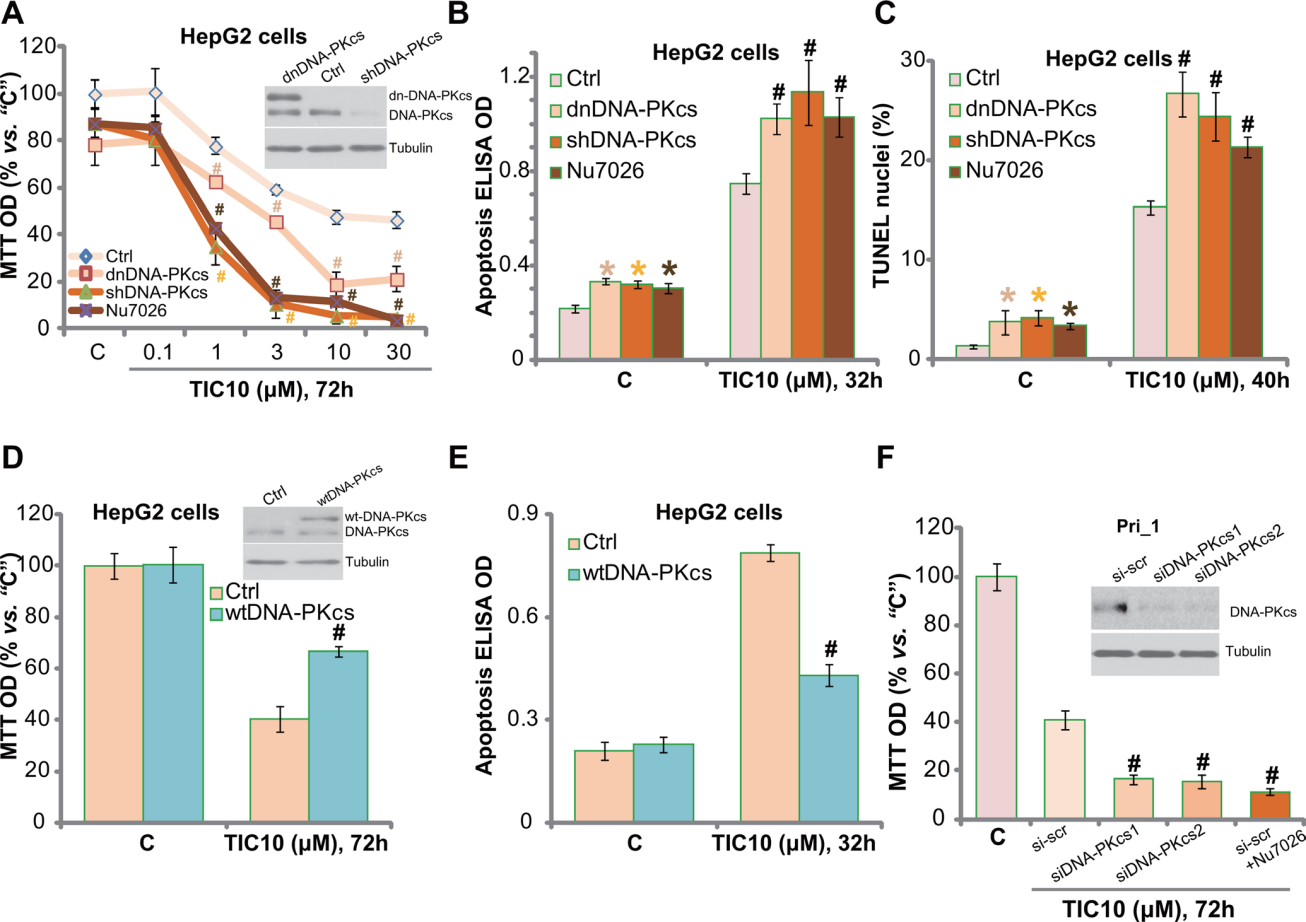
DNA-PKcs could be a primary resistance factor of TIC10 in HCC cells HepG2 cells, expressing dominant negative DNA-PKcs (“dnDNA-PKcs”, Flag-tagged), DNA-PKcs shRNA (“shDNA-PKcs”), wild-type DNA-PKcs (“wtDNA-PKcs”, Flag-tagged), or the parental control HepG2 cells (“Ctrl”), were treated with applied concentration of TIC10, or together with the DNA-PKcs inhibitor Nu7026 (10 μM), cells were further cultured in the conditional medium for applied time; Cell proliferation was tested by MTT assay (**A** and **D**); Cell apoptosis was tested by the Histone DNA ELISA assay (**B** and **E**) or TUNEL staining assay (**C**); Expression of DNA-PKcs (both wild-type and mutant) and Tubulin (loading control) were shown (A and D, upper panels). The primary human HCC cells (“Pri_1”), transfected with DNA-PKcs siRNAs (“-1/−2”) or scramble siRNA (“si-scr”), were treated with TIC10 (10 μM) or plus Nu7026 (10 μM) for indicated time; Cell proliferation was tested by MTT assay (**F**); Expressions of DNA-PKcs and Tubulin (loading control) were shown (F, upper panel). Experiments in this figure were repeated for three times, with similar results obtained. *n* = 5 for each repeat. Bars stand for mean ± SD *p < 0.05 vs. group “C”. ^#^*p* < 0.05 vs. “Ctrl” (A–E) or “si-scr” (F).

Next, we tested the potential resistance factor of TIC10 by focusing on DNA-PKcs. We utilized previous strategies [5]. The dominant negative mutant DNA-PKcs (T2609A) or DNA-PKcs shRNA was introduced into the HepG2 cells, and via selection, stable cell lines were established [5]. Western blotting assay results confirmed DNA-PKcs mutation or knockdown in above stable cells (Figure 3A, upper panel). Significantly, TIC10-induced proliferation inhibition, or MTT OD reduction, was potentiated in DNA-PKcs-mutated or -silenced HepG2 cells (Figure 3A). Likewise, Nu7026, a known DNA-PKcs inhibitor [36], also intensified TIC10′s cytotoxicity against HepG2 cells (Figure 3A). The IC-50 of TIC10, or the concentration that inhibited 50% of cell proliferation, decreased from 8.32 ± 0.73 μM to less than 1.0 μM when combined with Nu7026 or DNA-PKcs silence (Figure 3A). TIC10 (10 μM)-induced HepG2 cell apoptosis was also significantly augmented with DNA-PKcs silence, mutation or inhibition (Figure 3B and 3C). These results imply that DNA-PKcs could be a primary resistance factor of TIC10 in HCC cells. Notably, DNA-PKcs silence, mutation or inhibition only moderately induced proliferation inhibition and apoptosis in HepG2 cells (Figure 3A–3C), which were consistent with our previous findings [33].

Based on the above results, we would propose that DNA-PKcs over-expression may decrease TIC10′s cytotoxicity in HCC cells. Therefore, the wild-type DNA-PKcs (“wtDNA-PKcs”) construct (from our previous study [5]) was introduced to the HepG2 cells, and stable cell line was again established. Western blotting assay results in Figure 3D (upper panel) confirmed the expression of the wtDNA-PKcs (Flag-tagged) in the stable cells. Remarkably, DNA-PKcs over-expression in HepG2 cells indeed largely attenuated TIC10-induced proliferation inhibition (Figure 3D) and apoptosis (Figure 3E). These results further confirm the function of DNA-PKcs against TIC10 in HepG2 cells.

To study DNA-PKcs's effect on TIC10 in the primary HCC cells, siRNA strategy was applied to transitorily knockdown DNA-PKcs in the primary human HCC cells (“Pri_1”). The two applied DNA-PKcs siRNAs (see Method) efficiently downregulated DNA-PKcs in the primary cancer cells (Figure 3F, upper panel). TIC10 (10 μM)-induced cytotoxicity, tested by MTT OD reduction (Figure 3F), was also potentiated with DNA-PKcs siRNA knockdown. Further, co-treatment with Nu7026 similarly facilitated TIC10-induced anti-proliferative activity in the primary HCC cells (Figure 3F). Collectively, these results imply that DNA-PKcs could be a primary resistance factor of TIC10 in HCC cells.

### DNA-PKcs counteracts TIC10-induced Foxo3p nuclear translocation

**Figure 4:**
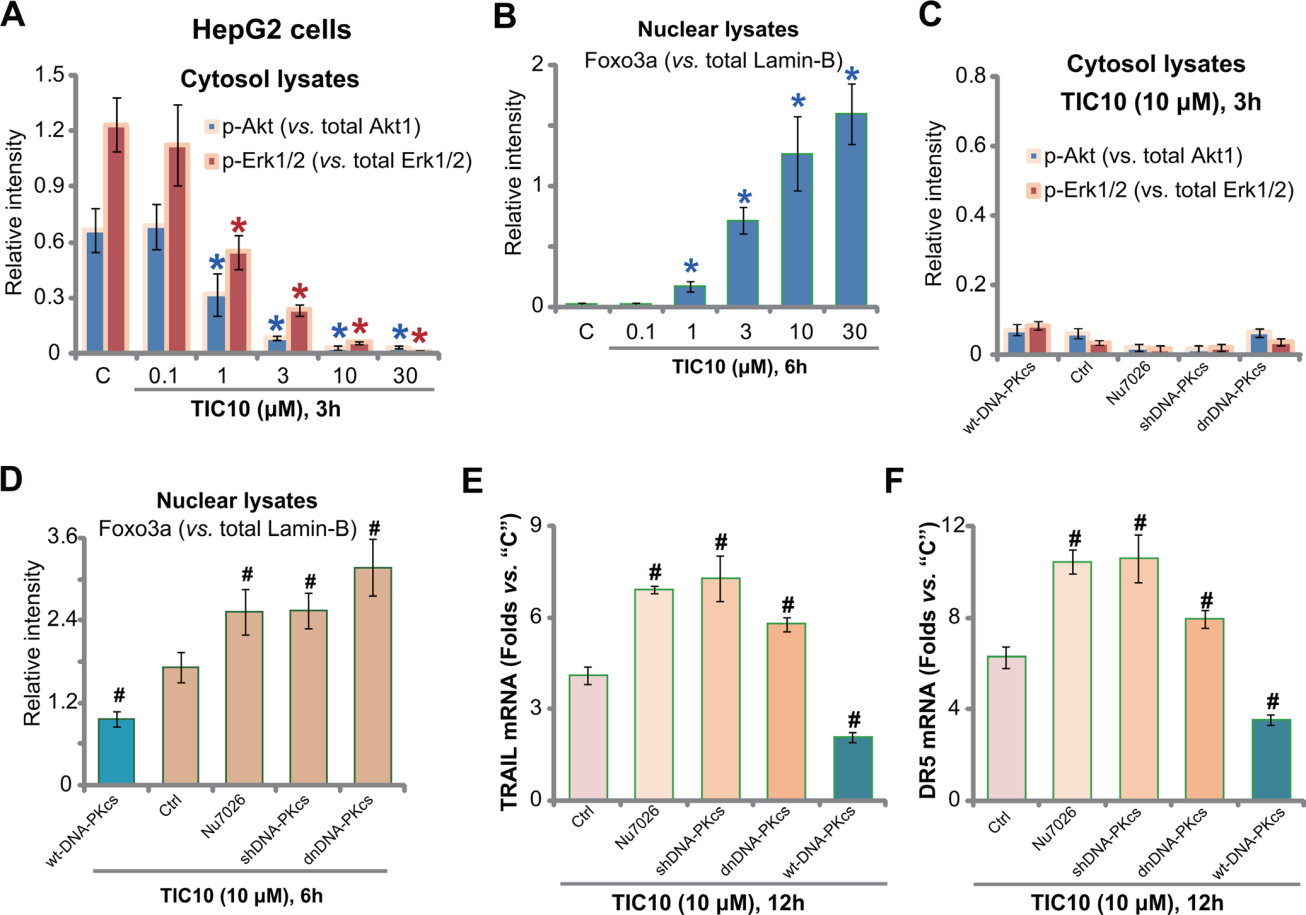
DNA-PKcs counteracts TIC10-induced Foxo3p nuclear translocation HepG2 cells were treated with applied concentration of TIC10 (0.1-30 μM), cells were then cultured in conditional medium for applied time; Expressions of listed proteins in cytosol (**A**) or nuclear (**B**) fraction lysates were tested by Western blotting assay, and five sets of repeated blots were quantified (A-B). HepG2 cells, expressing dominant negative DNA-PKcs (“dnDNA-PKcs”), DNA-PKcs shRNA (“shDNA-PKcs”), wild-type DNA-PKcs (“wtDNA-PKcs”), or the parental control cells (“Ctrl”), were treated with TIC10 (10 μM), or together with Nu7026 (10 μM), cells were further cultured in the conditional medium for applied time; Expressions of listed proteins in cytosol (**C**) or nuclear (**D**) fraction lysates were tested by Western blotting assay, and five sets of repeated blots were quantified (C-D); TRAIL (**E**) and DR5 (**F**) mRNA expression was also tested. *p < 0.05 vs. group “C”. ^#^*p* < 0.05 vs. “Ctrl” cells.

It is known that TIC10 inhibits Akt and Erk signalings, causing Foxo3a nuclear translocation to promote TRAIL and DR5 transcription [10, 12, 35]. The above signalings were tested in TIC10-treated HepG2 cells. Quantified Western blotting assay results in Figure 4A (Summarizing 5 sets of repeated blots) showed that TIC10 dose-dependently inhibited activation of Akt (p-Akt Ser-473) and Erk (p-Erk1/2 Tyr202/Thr204) in HepG2 cells. Consequently, Foxo3a was translocated to nuclei (Figure 4B). Notably, DNA-PKcs inhibition (by Nu7026), shRNA knockdown, dominant negative mutation, or over-expression didn't change TIC10-induced Akt and Erk inhibition (Figure 4C, summarizing 5 sets of repeated blots). Yet Foxo3p nuclear translocation was altered with above DNA-PKcs manipulations (Figure 4D, summarizing 5 sets of repeated blots). DNA-PKcs inhibition, knockdown or mutation facilitated TIC10-induced Foxo3a nuclear translocation (Figure 4D). Consequently, TIC10-induced expression of TRAIL and DR5 mRNAs was also increased (Figure 4E and F). Reversely, over-expression of DNA-PKcs decreased the amount of nuclear Foxo3a following TIC10 treatment (Figure 4D). TRAIL and DR5 mRNA expression was also inhibited (Figure 4E and 4F). Thus, in-activation of Akt and Erk by TIC10 causes Foxo3a nuclear translocation. DNA-PKcs apparently negatively regulates this process by inhibiting Foxo3a translocation. Reversely, DNA-PKcs interfere thus facilitates TIC10-induced Foxo3a translocation to promote TRAIL/DR5 transcription and cell apoptosis.

### Nu7026 facilitates TIC10-induced anti-HepG2 tumor activity *in vivo*

**Figure 5:**
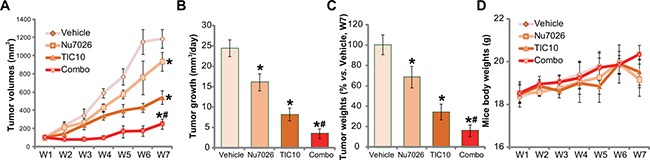
Nu7026 facilitates TIC10-induced anti-HepG2 tumor activity *in vivo* HepG2 bearing nude mice (*n* = 10 for each group) were administrated daily with TIC10 (30 mg/kg, oral administration) and/or Nu7026 (50 mg/kg, intraperitoneal injection, *i.p*.) for a total of 21 days; Weekly tumor volumes (in mm^3^) (**A**) and mice body weights (in gram) (**D**) were shown; Tumor daily growth was also calculated (**B**). At the end of the experiments (“W7”), tumors were isolated and weighted (**C**). Bars stand for mean ± SD. “W” stands for Week. *p < 0.05 vs. group “Vehicle”. ^#^*p* < 0.05 vs. TIC10 treatment only (A-C).

At last, we tested the potential anti-HCC activity of TIC10 *in vivo*. As described previously [5], a significant number of HepG2 cells were injected *s.c*. to the nude mice, and HepG2 tumor xenograft model was established. Tumor growth curve results in Figure 5A demonstrated that oral administration of TIC10 (30 mg/kg, daily) [13, 16, 37] efficiently inhibited HepG2 tumor growth in the nude mice. Remarkably, co-administration of Nu7026 (50 mg/kg, intraperitoneal injection, *i.p*.) [36, 38] potentiated TIC10-induced anti-HepG2 tumor activity (Figure 5A). TIC10 plus Nu7026 co-administration resulted in profound HepG2 tumor growth suppression, and the combined anti-tumor activity was more potent than each single treatment (Figure 5A). Treatment with Nu7026 alone only induced minor inhibition of HepG2 tumors (Figure 5A). Estimated daily tumor growth (in mm^3^ per day) was also dramatically suppressed by the co-administration, which was again more potent than each single administration (Figure 5B). Further, the weight of tumors in the co-administration mice was much lower than the vehicle control mice (Figure 5C), although each single treatment also decreased the tumor weights (Figure 5C). It should be noted that the above single or combined treatment didn't significantly change the mice body weight (Figure 5D). We also failed to observe apparent toxicities in the mice. Thus, these mice were well-tolerated to the above single or combination treatment regimen.

## DISCUSSION

Recent studies have proposed a chemo-resistance function of DNA-PKcs in cancer cells. Wu *et al*., showed that DNA-PKcs inhibition sensitized anti-colorectal cancer cell activity by WAY-600, a mTOR kinase inhibitor [39]. Hu *et al*., demonstrated that DNA-PKcs-mediated Akt activation acted as a key chemoresistance factor of gemcitabine in pancreatic cancer cells [40]. Very recently, Zhen *et al*., showed that DNA-PKcs could be a primary resistance factor of salinomycin in osteoblastoma cells [38]. DNA-PKcs is also important for the chemoresistance of human cervical carcinoma cells [41]. Our previous studies have shown that DNA-PKcs is over-expressed in multiple human HCC tissue [33].

There are several mechanisms to explain DNA-PKcs-mediated oncogenic behaviors. For example, studies have proposed the requirement of DNA-PKcs in mediating Akt activation in cancer cells [25, 40, 42, 43]. However, our previous study has shown that DNA-PKcs interference didn't change Akt activation in HCC cells [33]. Literatures also have implied that DNA-PKcs repairs damaged DNA to inhibit cell apoptosis [44–46]. In the current study, we proposed a novel mechanism of DNA-PKcs-induced chemoresistance: DNA-PKcs counteracts TIC-10-induced Foxop3 nuclear translocation, thus inhibiting TRIAL/DR5 transcription. We discovered that inhibition, shRNA knockdown or dominant negative mutation of DNA-PKcs facilitated TIC-10-induced Foxo3a nuclear translocation, TRAIL/DR5 expression and cell apoptosis. Reversely, exogenous DNA-PKcs over-expression inhibited above actions by TIC10. *In vivo*, Nu7026 co-administration potentiated TIC10-induced anti-HepG2 tumor activity in nude mice. Thus, DNA-PKcs inhibition should be a fine strategy to sensitize TIC10′s anti-cancer activity.

Interestingly, we showed that TIC10 was non-cytotoxic to HL-7702 hepatocytes and primary adult human hepatocytes. These results are consistent with recent findings showing no or minor cytotoxicity of this compound to non-cancerous normal cells [10, 12, 16]. This could be due to several reasons. For example, we have shown that the basal Akt and Erk activation was relatively high in HCC cells [33]. Yet, Akt and Erk activation level was extremely low in the above hepatocytes. Therefore, these hepatocytes may not be targeted by TIC10 due to low basal Akt/Erk activation. As a matter of fact, we failed to detect significant TRAIL expression in TIC10-treated above hepatocytes. Second, TIC10-induced TRAIL expression was shown to only kill cancerous cells, but not normal cells [9]. Nevertheless, the selective cytotoxicity of this compound to cancerous cells is promising for the clinical aspec.

Recent studies have demonstrated that molecularly-targeted therapies could benefit a number of HCC patients [6, 8]. The results of this preclinical study suggest that TIC10 could be further tested as a promising anti-HCC agent, alone or together with DNA-PKcs inhibitors.

## MATERIALS AND METHODS

Culture of established cell lines. Cultures of established human HCC cell lines, HepG2 and Huh-7, as well as the human HL-7702 hepatocytes were described previously [5].

Culture of primary cells. As described [5, 47], surgery-isolated fresh HCC tissue specimens of two written-inform consent patients, both administered at Wuxi People's Hospital (Wuxi, China), were subjected to collagenase I (Sigma) digestion. Primary cancer cells were then pelleted, washed, and re-suspended in the medium described [5, 47]. The two cell lines were named as “Pri_1” and “Pri_2”. Human primary adult hepatocytes were purchased from the Cell Bank of Fudan University (Shanghai, China). The hepatocytes were derived from the liver of a partial hepatectomy patient. The primary hepatocytes were maintained in the same primary cell culture medium [5]. Experiments and protocols requiring human tissues were approved by the Internal Review Board (IRB) and Ethics Committee of all authors’ institutions, and were according to the principles expressed in the Declaration of Helsinki.

Chemicals, reagents and antibodies. TIC10 was purchased by Selleck (Shanghai, China). The caspase-3 specific inhibitor z-DEVD-fmk, the caspase-8 specific inhibitor z-IETD-fmk, and the general caspase inhibitor Z-VAD-fmk were purchased from Sigma Chemicals (Louis, MO). Nu7026 was purchased from Calbiochem (San Diego, CA). All the antibodies were obtained from Cell Signaling Tech (Danvers, MA) [5, 25, 48].

MTT assay of cell proliferation. Cell proliferation was tested by the 3-(4,5-dimethylthiazol-2-yl)-2,5-diphenyltetrazolium bromide (MTT) assay as described [47].

siRNA knockdown of DNA-PKcs in primary cells. The two DNA-PKcs siRNAs, “siDNA-PKcs-1”, 5′-AGGGCCAAGCTGTCACTCT-3′ and “siDNA-PKcs-2”, 5′-GAUCGCACCUUACUCUGUUTT-3′, were described early [5]. SiRNA (200 nM, 24 hours) transfection was performed via Lipofectamine 2000 reagent (Invitrogen, Karlsruhe, Germany). After transfection, DNA-PKcs knockdown in the primary HCC cells was verified by Western blotting assay.

DNA-PKcs shRNA and stable cell selection. DNA-PKcs knockdown via lentiviral DNA-PKcs shRNA (Seq-1, see our previous study [5]) and puromycin-mediated stable cell selection were described previously [5]. Knockdown of DNA-PKcs in stable cells was always verified by Western blotting assay.

DNA-PKcs mutation or overexpression. The pSV2 neo Flag plasmid with the wild-type (“wtDNA-PKcs”) or dominant negative mutant DNA-PKcs (T2609A, “dnDNA-PKcs”) was described early [5]. The construct was transfected to HepG2 cells by Lipofectamine 2000 [25]. Stable HepG2 cells were subjected to G418 (100 μg/mL) selection for 10 days. DNA-PKcs expression in stable cells was verified by Western blotting assay.

RNA extraction and real-time PCR. RNA extraction and quantitative real time-PCR assay (using an ABI Prism 7500 Fast Real-Time PCR system) were performed as previously described [5, 49, 50]. After amplification, melt curve analysis was applied to calculate product melting temperature. *GAPDH* gene was chosen as the reference gene, and the 2^−ΔΔCt^ method was applied to quantify targeted mRNA change within samples [49, 50]. The primers for human *TRAIL*: forward, 5′-CCTGGGCGATAAAGTGAGAT-3′ and reverse, 5′-GGCCCAGCTGTATGTTGTCT-3′ [51]. 3′. For human *death receptor-5* (*DR5*): forward, 5′-AAGACCCTTGTGCTCGTTGT-3′; and reverse, 5′-AGGTGGACACAATCCCTCTG-3′ [52].

*In vivo* anti-tumor efficiency assay. As reported early, HepG2 cells in logarithmic growth phase were injected subcutaneously (*s.c*.) into the right flanks of female nude mice (6–7 weeks old, 18-19 grams). When tumors reached around 100 mm^3^ (probably 2-3 weeks), mice were randomized into four groups (10 mice per group). Treatment was described in the text. Tumor volume was calculated through the established formula as reported [5, 53, 54].

For “Clonogenicity” assay of cell proliferation, [H^3^] Thymidine incorporation assay of cell proliferation, Caspase-3/−8 activity assay, Histone DNA-ELISA assay of cell apoptosis, TUNEL staining of apoptosis assay, and Western blotting assay, please refer to our previous studies [5, 47, 50, 55–57]. When testing nuclear proteins, the nuclei of HCC cells were isolated by the nuclei Isolation kit of Sigma.

Statistical analysis. Data were presented as mean ± standard deviation (SD). Statistics were analyzed by one-way ANOVA followed by a Scheffe’ and Tukey Test (SPSS 15.0). Significance was chosen as *p* < 0.05. IC-50 was calculated by the SPSS software.
